# A Comparison of the Variable J and Carbon-Isotopic Composition of Sugars Methods to Assess Mesophyll Conductance from the Leaf to the Canopy Scale in Drought-Stressed Cherry

**DOI:** 10.3390/ijms21041222

**Published:** 2020-02-12

**Authors:** Giovanni Marino, Matthew Haworth, Andrea Scartazza, Roberto Tognetti, Mauro Centritto

**Affiliations:** 1 National Research Council of Italy - Institute of Sustainable Plant Protection (CNR - IPSP), Via Madonna del Piano 10, 50019 Sesto Fiorentino (FI), Italy; matthew.haworth@ipsp.cnr.it (M.H.); mauro.centritto@cnr.it (M.C.); 2 National Research Council of Italy—Research Institute on Terrestrial Ecosystems (CNR–IRET), Via Moruzzi 1, 56124 Pisa, Italy; andrea.scartazza@cnr.it; 3 Department of Agricultural, Environmental and Food Sciences - University of Molise, Via Francesco De Sanctis, 86100 Campobasso, Italy; tognetti@unimol.it; 4 The EFI Project Centre on Mountain Forests (MOUNTFOR), Edmund Mach Foundation, 38010 San Michele all’Adige (TN), Italy; 5 CNR-Eni Research Center “Acqua”, Research Center Metapontum Agrobios, 750125 Metaponto, Italy

**Keywords:** *Prunus avium* L., photosynthesis, transport conductance, ^13^C stable isotope, water deficit, photosystem II quantum efficiency, sugars

## Abstract

Conductance of CO_2_ across the mesophyll (*G*_m_) frequently constrains photosynthesis (*P*_N_) but cannot be measured directly. We examined *G*_m_ of cherry (*Prunus avium* L.) subjected to severe drought using the variable *J* method and carbon-isotopic composition (*δ*^13^C) of sugars from the centre of the leaf, the leaf petiole sap, and sap from the largest branch. Depending upon the location of the plant from which sugars are sampled, *G*_m_ may be estimated over scales ranging from a portion of the leaf to a canopy of leaves. Both the variable *J* and *δ*^13^C of sugars methods showed a reduction in *G*_m_ as soil water availability declined. The *δ*^13^C of sugars further from the source of their synthesis within the leaf did not correspond as closely to the diffusive and C-isotopic discrimination conditions reflected in the instantaneous measurement of gas exchange and chlorophyll-fluorescence utilised by the variable *J* approach. Post-photosynthetic fractionation processes and/or the release of sugars from stored carbohydrates (previously fixed under different environmental and C-isotopic discrimination conditions) may reduce the efficacy of the *δ*^13^C of sugars from leaf petiole and branch sap in estimating *G*_m_ in a short-term study. Consideration should be given to the spatial and temporal scales at which *G*_m_ is under observation in any experimental analysis.

## 1. Introduction

The availability of carbon dioxide (CO_2_) for the carboxylation of ribulose-1,5-bisphosphate (RuBP) inside the chloroplast frequently limits the rate of photosynthesis (*P*_N_). The chloroplastic [CO_2_] (*C*_c_) is lower than atmospheric [CO_2_] (*C*_a_) largely due to resistance in the diffusion of CO_2_ experienced at the stomata and mesophyll layers [1,2,3]. Measurement of the diffusion of water vapour from the internal leaf air-spaces to the external atmosphere allows the calculation of stomatal conductance (*G*_s_) and has permitted the characterisation of *G*_s_ responses to factors such as drought or *C*_a_ [3,4,5,6,7]. It is not possible to directly measure the transport of CO_2_ across the mesophyll layer to the site of carboxylation within the chloroplast envelope (termed mesophyll conductance: *G*_m_); therefore, a number of methodologies have been developed to approximate *G*_m_. Such quantification of *G*_m_ has demonstrated the importance of the movement of CO_2_ across the mesophyll layer to *P*_N_ and plant acclimation to changing growth conditions [1,3,8,9]. Indeed, the physical [10] and biochemical [11] factors influencing *G*_m_ are key attributes in the development of more productive and/or drought-tolerant crops [12,13]. However, the methods used to estimate *G*_m_ all involve certain assumption and aspects susceptible to error [14]. Moreover, as some methods require sensitive equipment in addition to standard gas exchange [15] or extended periods of measurement [16] they are not suited to use in the field. Here, we utilised the ‘variable *J*’ method involving simultaneous leaf gas exchange and chlorophyll fluorescence (Chl-Flr) [17] alongside analysis of the carbon isotopic composition (*δ*^13^C) of recently synthesised sugars [18,19] to characterise *G*_m_ in cherry (*Prunus avium*) subject to drought.

The assimilation of CO_2_ during photosynthesis creates a diffusion gradient between the chloroplast and the internal leaf air-spaces; however, the conductance of CO_2_ across the mesophyll is highly complex, involving gaseous and aqueous phases, the biochemistry of the mesophyll and physical resistances [14,20,21]. The physical structure of the mesophyll plays a major role in *G*_m_ [22]; species with increased surface area [10] and lower distances between the air-space and chloroplasts in the internal leaf air-spaces [23] tend to exhibit higher *G*_m_. Mesophyll conductance has also been shown to change with leaf structure and during leaf expansion [24]. The abundance and activity of carbonic anhydrases and cooporins (a sub-set of aquaporin proteins) involved in the transport of CO_2_ have been shown to determine rapid *G*_m_ variation [11,25]. Stomatal and mesophyll conductance generally respond in tandem to a change in growth conditions such as drought [1,7,19,26]. Stomatal conductance may affect *G*_m_ through its action upon the concentration of CO_2_ in the internal leaf air-space (*C*_i_) [3,27,28,29]. However, the apparent correlation between *G*_s_ and *G*_m_ may also be the result of artefacts associated with CO_2_ released via photorespiration and mitochondrial respiration. Despite the expansion of research into the movement of CO_2_ from the internal leaf air-space to the chloroplast in relation to changes in environmental conditions, *G*_m_ has been characterised as being fixed [30,31], dynamic [28,32], or a ‘flux-weighted quantity’ [33]. For example, the variations in *G*_m_ observed with changes in CO_2_ [28] or light [32,34] may be the result of artefacts associated with the calibration of electron transport and re-capture of CO_2_ as photorespiration varies [31,33,34]. The estimation of *G*_m_ using different techniques may reduce some of these ambiguities due to the contrasting strengths and weaknesses of each methodology [2,14,20].

Mesophyll conductance can be determined by simultaneous analysis of leaf gas exchange and Chl-Flr (the variable J and constant J methods: 2), curve fitting analysis of the *P*_N_–*C*_i_ response [16,30], measurement of *P*_N_ under different [O_2_] [35], and carbon isotope discrimination [15,36]. All of these protocols rely upon measurement of leaf gas exchange, and so are not truly independent of one another (a comprehensive review is available in [20]). The estimation of *G*_m_ from gas exchange techniques and especially from *P*_N_–*C*_i_ response curves requires the removal of diffusion leaks [37], as well as sufficient time to not only perform the response curve but also remove stomatal limitations [3], which may make this approach less favourable in the field [6,38]. Modification of [O_2_] also requires cylinders of O_2_ and N_2_ along with the facility to mix these gases, making it very difficult for measurement of *G*_m_ outside the laboratory. The variable *J* approach [17,39] is the most widely used method to measure *G*_m_ due to the incorporation of Chl-Flr capabilities in most commercial plant photosynthesis gas exchange systems, meaning measurements can be conducted within a self-contained piece of equipment (a factor that is of considerable importance while working in the field). The variable *J* method estimates *G*_m_ by utilising gas exchange and Chl-Flr measurements to calculate *C*_c_ [17]. However, uncertainties associated with leaks [40], variations in photorespiration, respiration in the light and electron sinks [33,34], accurate determination of the maximum fluorescence [41], and sensitivity of the method in species with high *G*_m_ (where the differences between *C*_a_ and *C*_c_ are less apparent) [2] may limit the effectiveness of the variable *J* method in gauging *G*_m_.

Photosynthetic uptake and assimilation of CO_2_ discriminate against the heavier ^13^C-isotope. This results in non-structural carbohydrates and plant structural tissues being enriched in the lighter ^12^C-isotope. As stomata close during drought, the discrimination against the uptake of ^13^C declines and the *δ*^13^C of leaves become enriched in the heavier isotope [42]. Combining C-isotope discrimination with gas exchange parameters can allow estimation of *G*_m_ by comparison of the difference between the observed *δ*^13^C and the theoretically expected C-isotopic composition if *G*_m_ were infinite [15,36]. The C-isotopic composition of air passing over a leaf surface in a gas exchange system can be measured online using an isotope ratio mass spectrometer [43] or tuneable diode laser absorption spectroscopy [44]. This can allow “instant” estimation of *G*_m_ in response to a change in cuvette conditions. However, this method requires sufficiently sensitive measurement of the isotopic composition of the air, and is not yet suited for the analysis of *G*_m_ in the field [20]. It is also possible to estimate *G*_m_ on the basis of the *δ*^13^C of recently synthesised sugars, giving a representative approximation of C-isotopic discrimination over a period of hours (in the case of sugars) to days/weeks (in the case of carbohydrates stored as starch) [18,45]. This approach is suited to field-based analysis of *G*_m_, as leaves can be flash frozen after gas exchange analysis to enable the extraction of sugars later in the laboratory [8,19]. This technique has been utilised to assess *G*_m_ on a wider spatial scale by analysing the *δ*^13^C of sugars in the sap of leaf petioles and whole branches [46], or gas exchange of a whole branch enclosed within a bag [47]. The δ^13^C of sugars from the sap of larger branches will in effect integrate greater spatial and temporal variation in *G*_m_ [46].

Given the prominent role played by *G*_m_ in *P*_N_ under changing environmental conditions, as well as the importance of accurate measurement of temporal and spatial variations in the transport of CO_2_ across the mesophyll integrated at whole leaf and/or branch level, we assessed *G*_m_ in cherry subject to sharp drought stress using the variable *J* and C-isotopic composition of recently synthesised sugar approaches. This study aimed to (i) determine whether instantaneous measurement of *G*_m_ using the variable *J* method is comparable to the longer term integration of *G*_m_ derived from the C-isotopic composition of recently synthesised sugars; (ii) assess whether it is feasible to quantify *G*_m_ over wider spatial scales through analysis of the C-isotopic composition of sugars in the leaf petiole and branch sap (i.e., to give a wider indication of whole leaf or average canopy *G*_m_), particularly given the necessity of conducting instantaneous point measurements of gas exchange on a restricted leaf area; (iii) examine *G*_m_ in relation to the leaf position along a branch using the variable *J* method to characterise spatial variations in *G*_m_, and whether this corresponds to *G*_m_ calculated from the *δ*^13^C of sugars derived from the leaf petiole and branch sap; and (iv) discuss the relative merits and weaknesses of the variable *J* and *δ*^13^C of recently synthesised sugar methods for the calculation of *G*_m_, as well as the applicability of these methods to future studies of *G*_m_ from the leaf to the canopy scale.

## 2. Results

**Figure 1:**
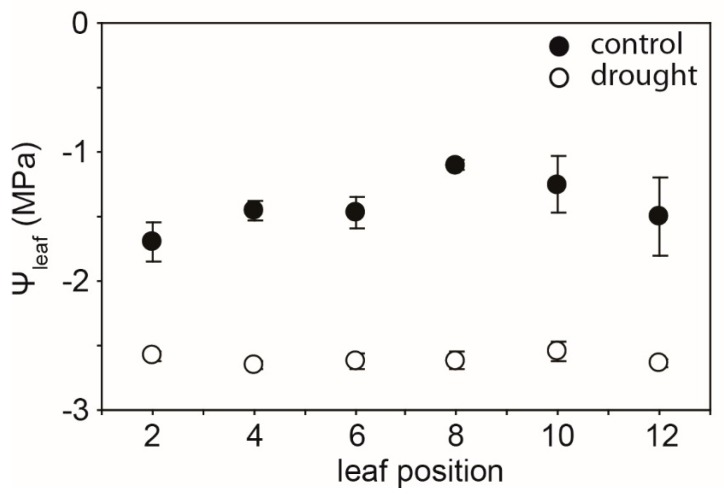
Leaf water potential (*Ψ*_leaf_) of leaves along the largest branch of well-watered control and drought-stressed cherry after 5 days. Symbols represent the mean of six plants. Error bars indicate one standard error either side of the mean.

**Figure 2:**
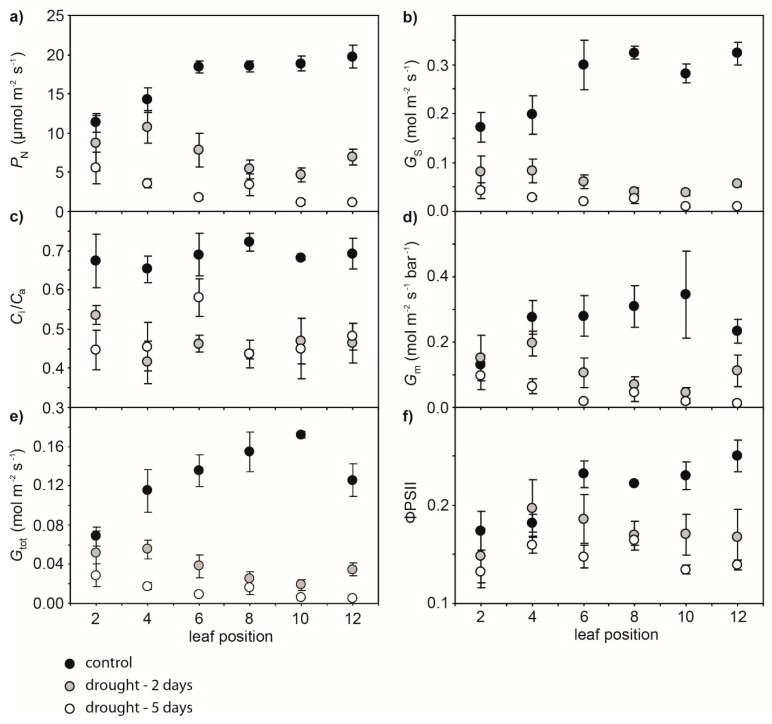
Gas exchange and chlorophyll fluorescence (Chl-Flr) parameters of the leaves along the largest branch of drought-stressed cherry trees after 2 days (grey fill) and well-watered control (black fill) and drought-stressed (white fill) cherry trees after 5 days: (**a**) photosynthesis (*P*_N_), (**b**) stomatal conductance (*G*_s_) of water vapour, (**c**) the ratio of the atmospheric [CO_2_] (*C*_a_) to the concentration of CO_2_ (*C*_i_) within the internal leaf air-space, (**d**) mesophyll conductance to CO_2_ (*G*_m_) calculated using the variable *J* method, (**e**) the total conductance to CO_2_ (*G*_tot_), and (**f**) the actual quantum efficiency of photosystem II (ΦPSII). Symbols represent the mean of six plants. Error bars indicate one standard error either side of the mean.

**Figure 3:**
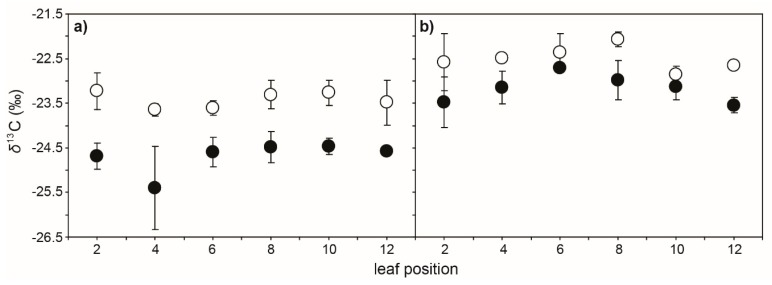
Carbon isotope composition (*δ*^13^C) of leaf-soluble sugars (**a**) and leaf petiole sap (**b**) along the largest branch of control (black fill) and drought-stressed (white fill) cherry after 5 days. Symbols represent the mean of six plants. Error bars indicate one standard error either side of the mean.

**Figure 4:**
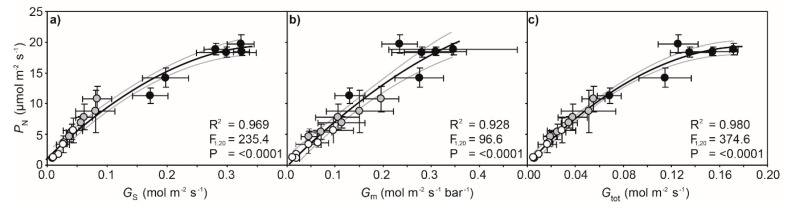
The relationship of photosynthesis to stomatal conductance (*G*_s_) to CO_2_ (**a**); mesophyll conductance to CO_2_ (*G*_m_) calculated using the variable *J* method (**b**); and total conductance to CO_2_ (*G*_tot_) (**c**) of drought-stressed cherry trees after 2 days (grey fill), and well-watered control (black fill) and drought-stressed (white fill) cherry trees after 5 days. Symbols represent the mean of six plants. Error bars indicate one standard error either side of the mean. Non-linear regression was used to assess the significance of any relationship. The black line indicates a logarithmic best-fit line and the two grey lines either side indicate the 95% confidence intervals of the mean.

Drought resulted in progressive declines in the water potential of the leaf (*Ψ*_leaf_) values of cherry leaves as soil dried over the 5 day experimental period. The reduction in *Ψ*_leaf_ after 5 days of soil drying was lowest in the leaves near the apex of the branch (leaf positions 2 and 8 showed 51.9% and 137.8% reductions, respectively, in *Ψ*_leaf_ after 5 days) (Figure 1). Photosynthesis and *G*_s_ showed similar reductions as drought progressed. The impact of 2 days of soil drying was less apparent on *P*_N_ and *G*_s_ of leaves nearer the branch apex, although in well-watered control plants, *P*_N_ and *G*_s_ values in the second and fourth leaves were lower than those observed in the more basal leaf positions (Figure 2a,b). Stomatal closure associated with lower *G*_s_ resulted in a reduction in the *C*_i_/*C*_a_ ratio (Figure 2c) and an increase in *δ*^13^C of both leaf and leaf petiole sap sugars (Figure 3). Mesophyll conductance measured using the variable *J* method (Figure 2d) and total conductance to CO_2_ (*G*_tot_) (Figure 2e) exhibited similar reductions to *P*_N_ and *G*_s_ along the stem as a result of soil drying. The actual quantum efficiency of PSII (ΦPSII) declined as drought developed, with the effect being most pronounced in the lower leaves in the branch from position 10 to 12 (Figure 2f). Rates of *P*_N_ in the cherry plants after 2 and 5 days of soil drying were positively related to stomatal (Figure 4a), variable *J* mesophyll (Figure 4b), and total (Figure 4c) conductance to CO_2_.

**Figure 5:**
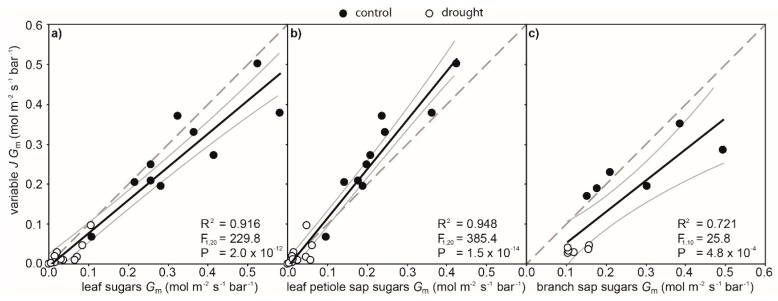
Correlations between mesophyll conductance (*G*_m_) to CO_2_ calculated using the variable *J* method and *G*_m_ calculated using the carbon isotopic composition of sugars collected from the leaf (**a**), leaf petiole sap (**b**), and branch sap (**c**) of well-watered control (black fill) and drought-stressed (white fill) cherry trees after 5 days. Linear regression was used to assess the significance of any relationship. The black line indicates the line of best fit, and the two grey lines either side indicate the 95% confidence intervals of the mean. The broken grey line indicates a hypothetical 1:1 relationship between *G*_m_ determined using the variable *J* method and those derived from the carbon isotopic composition of sugars.

**Figure 6:**
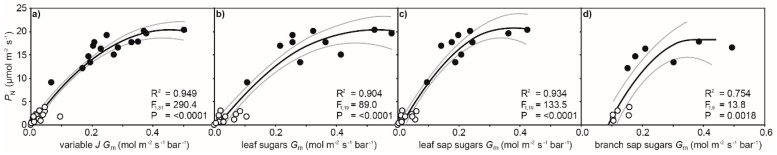
The relationship between photosynthesis (*P*_N_) and mesophyll conductance (*G*_m_) to CO_2_ calculated using the variable *J* method (**a**) and the carbon isotopic composition of sugars collected from the leaf (**b**), leaf petiole sap (**c**), and branch sap (**d**) of well-watered control (black fill) and drought-stressed (white fill) cherry trees after 5 days. Linear regression was used to assess the significance of any relationship. The black line indicates the line of best fit and the two grey lines either side indicate the 95% confidence intervals of the mean.

The *δ*^13^C mean values of sugars extracted from the leaf, leaf petiole sap, and branch sap of drought-stressed cherry seedlings after 5 days of stress were respectively 1.28, 0.67, and 0.98‰ ^13^C-enriched than their well-watered control counterparts (Figure 3 and Table 1), although leaf petiole sap showed a higher *δ*^13^C than the other sugar sources. Mesophyll conductance values determined using the *δ*^13^C of both leaf and leaf petiole sap sugars were 89% lower in leaves of drought-stressed than well-watered control cherry plants after 5 days. The *δ*^13^C of sugars derived from branch sap indicated that *G*_m_ values of drought-stressed cherry seedlings were 56% lower than those of control plants. The variable *J* method showed significant correlations to *G*_m_ estimated using the *δ*^13^C of recently synthesised sugars extracted from the leaf, leaf petiole sap, and branch sap (Figure 5). The variable *J* and C-isotopic composition of leaf sugars produced broadly comparable estimates of *G*_m_ (Figure 5a). Analysis of the sugars extracted from the sap of the leaf petiole of well-watered control plants produced slightly lower values of *G*_m_ than observed with the variable *J* method (Table 1 and Figure 5b). Values of average branch canopy *G*_m_ estimated from *δ*^13^C of sugars from the branch sap showed overlap between control and drought-stressed plants. This increased variability when determining average canopy *G*_m_ based on the *δ*^13^C of branch sap resulted in the weakest correlation compared to a branch-level average of *G*_m_ values determined using the variable *J* approach (Figure 5c). Photosynthesis was positively related to values of *G*_m_ determined by all of the approaches utilised in this study (Figure 6). The strongest correlation was found between *P*_N_ and *G*_m_ determined by the variable *J* method (Figure 6a). The weakest correlation was observed between branch average rates of *P*_N_ and average branch canopy *G*_m_ estimated from sugars in the branch sap (Figure 6d).

## 3. Discussion

This study has demonstrated the central role of *G*_m_ in determining the photosynthetic response of cherry trees to drought (Figure 4). The variable *J* and C-isotopic composition of sugars methods both indicated a reduction in *G*_m_ as soil water availability declined (Figure 5). If *G*_m_ is considered to act as a ‘flux-weighted’ quantity [33], this reduction in CO_2_ transport across the mesophyll is likely the result of reduced *P*_N_ lowering the uptake of CO_2_ (Figure 2) and stomatal closure (Figure 2b) leading to lower *C*_i_ (Figure 2e). Nonetheless, *G*_m_ was found to be a key constraint to *P*_N_ in cherry trees subject to drought (Figure 6), and manipulation of the biochemical and physical properties of the mesophyll layer [12] may enhance the productivity and drought tolerance of cherry trees.

Given that both methods produced broadly comparable values of *G*_m_ under well-watered control and drought-stressed conditions (Table 1), this strengthens the interpretation that lower transport of CO_2_ across the mesophyll in cherry limits *P*_N_ as soil water availability declines. However, it is worth noting that the variable J and δ^13^C of sugars methods to estimate *G*_m_ are not truly independent as both utilise the same gas exchange parameters (in particular *P*_N_, see Equations (1) and (4)) [17,18]. It is, therefore, reasonable to assume a degree of self-correlation in this instance; indeed, the closer correlation between *P*_N_ and variable J *G*_m_ (Figure 6a) may simply reflect the more prominent role of *P*_N_ in the formulae used to determine variable J *G*_m_. Nonetheless, differences were observed in *G*_m_ values determined using the δ^13^C of sugars method depending upon the source of the sugars (Figure 5). Although a drought-induced enrichment in ^13^C was evident in all of the analysed sugar components (Table 1), the differences in *δ*^13^C, and hence *G*_m_, are likely associated with temporal and spatial changes in C-isotopic discrimination during *P*_N_ and post-photosynthetic fractionation processes depending upon the sugar source [48,49]. The variable *J* and C-isotopic composition of sugars methods showed a high degree of correlation and correspondence between absolute values of *G*_m_ when leaf sugars were analysed (Figure 5a). As sugars in the leaf are those that have most recently been synthesised [50], these are likely to correspond most closely to the diffusive limitations to CO_2_ and C-isotopic discrimination reflected in the instantaneous measurements of gas exchange and Chl-Flr parameters utilised in the variable *J* method (Equation (3): [17]). The correlation between *G*_m_ values determined by the variable *J* and *δ*^13^C of leaf petiole sugars was slightly more significant than that produced from the *δ*^13^C of leaf sugars. However, the *δ*^13^C values of leaf petiole sugars were higher than sugars from the leaf or branch sap (Table 1) in both well-watered control and drought-stressed plants, indicating a higher proportion of the heavier ^13^C-isotope. The higher *δ*^13^C of sugars from the leaf petiole sap may reflect the impact of increased discrimination against sugars composed of the heavier isotope [51] during respiration or at other branch points of the metabolic pathways within the leaf, the methylerythritol pathway [52]. Discrimination in favour of sugars composed of the lighter ^12^C-isotope will progressively enrich the remaining pool of photosynthetic sugars destined for export from the leaves to the other parts of the plant. Previous studies have suggested that the diurnal rhythms of transitory starch accumulation and degradation may cause an isotopic partitioning between carbohydrates consumed within and exported from the leaf, thus contributing to the differences in isotopic signature between autotrophic and heterotrophic tissues [51]. Moreover, ^13^C enrichment in phloem sap could also be due to fractionation occurring during phloem loading or unloading, and to the contribution of starch or other heavier reserve compounds that may be hydrolysed and loaded in the phloem [53]. Furthermore, the leaf petiole sap was collected using the Scholander method and, therefore, represents a mixture of both xylem and phloem exudates composed of different apoplastic and membrane-filtered symplastic sap fractions [54]. Jachetta et al. [55] identified three distinct fractions successively released into the sap collected using the pressure chamber: a petiole-midrib fraction, a minor vein-cell wall fraction, and a mixed fraction composed of a combination of minor vein-cell wall fraction with an increasing proportion of membrane-filtered cell sap. Therefore, the isotopic signature of the leaf petiole sap could be the result of a combination of the different carbon sources and not simply recently synthesised photosynthate derived from the leaf. Nonetheless, post-photosynthetic ^13^C enrichment of the leaf petiole sugars may account for the reduced difference in *δ*^13^C values observed between control and drought-stressed plants, leading to a lower estimation of *G*_m_ compared to the other isotopic methods.

Average canopy *G*_m_ values produced from the *δ*^13^C of branch sap were less consistent, with a less robust correlation to average branch variable *J G*_m_ than the *δ*^13^C analyses of sugars from individual leaves and the sap of leaf petioles (Figure 5). The *δ*^13^C of branch sap sugars produced higher mean *G*_m_ in the drought-stressed plants than other approaches (Table 1). The increased variability in *G*_m_ from branch sugars may reflect the limitations of the “branch-scale average canopy” approach when applied to a short-term severe drought experiment such as the present study. Sugars in the branch phloem represent photosynthate from the canopy of leaves supported along that branch; as such, the *δ*^13^C of these sugars reflects wider temporal and spatial effects of C-isotope discrimination than those recorded by instantaneous measurement of gas exchange parameters [20,46]. Given that the development of drought stress in this study was fairly rapid and severe, it is likely that these impacts were not fully represented in the *δ*^13^C of sugars within the branch sap. Drought can affect sucrose loading and transport within the phloem and hydrolysis of starch reserves, contributing differently to the phloem carbon pool and to its isotopic signature [48,49]. Moreover, Bögelein et al. [56] demonstrated that the *δ*^13^C of leaf water-soluble compounds were more effective than the *δ*^13^C of phloem exudates in providing short-term physiological information. Hence, *δ*^13^C analysis of branch sap sugars may be useful in calculation of average canopy *G*_m_ in instances where growth conditions have remained stable for a sufficient period of time (days to weeks); indeed, average branch canopy *G*_m_ of control plants from the *δ*^13^C of branch sap sugar was statistically similar to values produced by the variable *J* method, consistent with the continuity of growth conditions for the well-watered control plants. Seasonal analysis of the average branch canopy *G*_m_ in three conifer species showed pronounced reductions in *G*_m_ during the summer months when water availability was lower [46]. Likewise, *δ*^13^C values of sugars in the stem sap of *Fagus sylvatica* were 6.6% greater in June than October, and also exhibited a 36.1% lower co-efficient of variance than the present study [49]. During drought, levels of non-structural carbohydrates increase within sap to facilitate osmoregulation [49,57]. Under drought conditions, as *P*_N_ declines, the increased sugars within the sap are likely derived from stored carbohydrates [49,58], and their isotopic signature would be mainly dependent on the environmental conditions when the CO_2_ was initially assimilated. Therefore, the release of previously stored carbohydrates in cambial tissues may constrain the effectiveness of the C-isotopic composition of sugars method in assessing average branch canopy *G*_m_ during short-term severe drought studies. The variations in *δ*^13^C of branch sap sugars can be dependent on a complex combination of photosynthetic and post-photosynthetic fractionation processes and also the interaction of plants with abiotic factors; therefore, this should be taken into account in evaluating *G*_m_ using phloem sap *δ*^13^C when environmental conditions are subjected to rapid change.

Average branch canopy *G*_m_ from the analysis of the *δ*^13^C of sugars methods may be enhanced by wider-scale screening of the gas exchange properties of the entire canopy over a longer duration of time. However, because of the complexities associated with the measurement of leaf gas exchange, it is not possible to continuously monitor a large number of individual leaves in a canopy over a long period. Bags may be used to record gas exchange over a whole branch [47]; however, these measurements reflect gas exchange at a single point in time. The use of infra-red thermography and/or spectroradiometry monitoring [59,60] may enable characterisation of gas exchange properties in the branch canopy over a sufficient duration. In conjunction with point or branch-scale measurements of *P*_N_ and *G*_s_, such remote sensing techniques may produce estimates of *P*_N_ that correspond more closely to the *δ*^13^C of the branch sap sugars to allow a more robust estimate of average branch canopy *G*_m_.

## 4. Materials and Methods

### 4.1. Plant Material and Growth Conditions

Twelve cherry (*Prunus avium*) saplings in 20 L pots filled with Amsterdam medium (a 9:1 mix of washed sand and compost) were grown for 4 months in a greenhouse at the Italian National Research Council. The plants were 2 years old and all around 1.5 m in height. The respective daily maximum and minimum air temperatures were 35 and 20 °C. To avoid any water and nutrient limitation, the seedlings were watered every other day to pot water capacity and fertilised once a week with Hoagland nutrient solution to supply nutrients at free access rates. The evening prior to measurement, the cherry seedlings were watered to pot water capacity and then half of the plants were allowed to dry, whereas the remaining six were watered to pot capacity each day over a 5 day period.

### 4.2. Gas Exchange and Fluorescence Measurements

**Figure 7:**
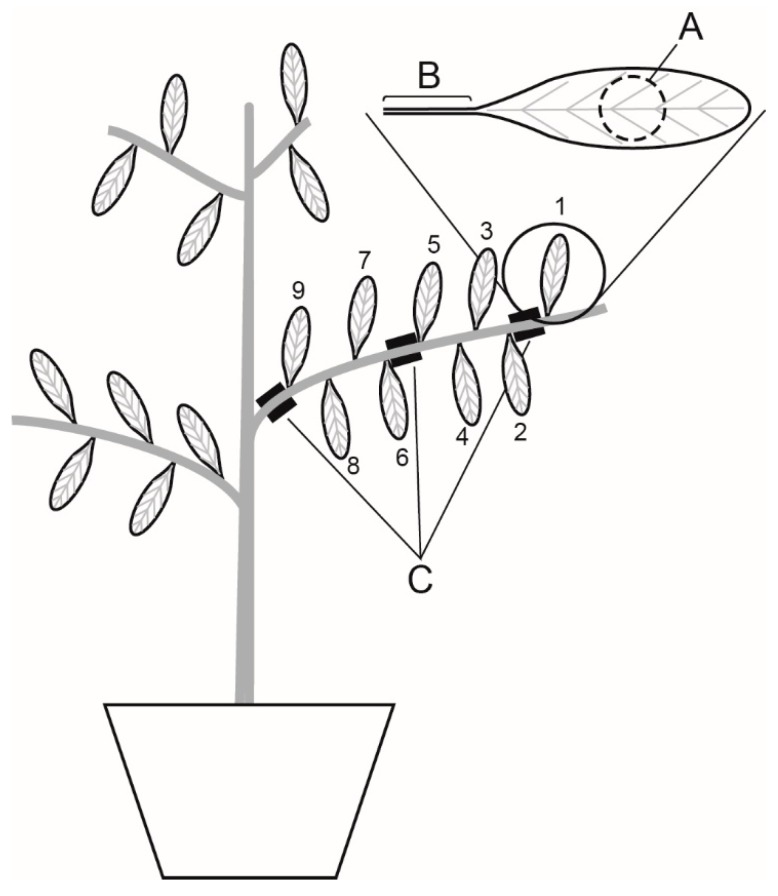
Schematic illustration of branch leaf position for gas exchange and Chl-Flr measurements and the location of sugars used for C-isotopic analysis from the leaf (point (**A**)), leaf petiole (point (**B**)), and branch sap (point (**C**)).

Simultaneous point measurements of *P*_N_, *G*_s_, *C*_i_, and the actual quantum efficiency of PSII (ΦPSII) were performed on the centre of each leaf using a LiCor Li6400XT fitted with a 6400-40 2 cm^2^ leaf cuvette (Li-Cor, Inc., Nebraska, USA) after 2 and 5 days. To minimize the possible effects of leaf development on *G*_m_ [24], the leaf in position 1 (Figure 7) was not analysed, and we ensured that the second youngest leaf (position 2) was at least 80% morphologically developed; lower values of *G*_s_ in leaf positions 2 and 4 may indicate that the leaves nearest the branch apex were not physiologically mature with respect to their counterparts in lower branch positions. The following environmental conditions were set in the cuvette: 1500 μmol m^−2^ s^−1^ photosynthetic photon flux density (PPFD: 10% blue and 90% red light), 400 ppm [CO_2_], leaf temperature of 25 °C, and a relative humidity of 45%. To reduce diffusive leaks through the chamber gasket, a supplementary gasket was added and the Li6400XT exhaust air was fed into the space between the chamber and the supplementary external gasket. To determine ΦPSII, the multi-phase fluorescence setting was used with an initial saturating pulse of 8000 μmol m^−2^ s^−1^ [41]. Point measurements of gas exchange and Chl-Flr were taken from leaves along the largest branch of six plants for each treatment, as illustrated in Figure 1. Mesophyll conductance (*G*_m_) was determined using the variable *J* method described by Harley, Loreto, Dimarco, and Sharkey [17]:

(1) $$G_{m}=\frac{P_{N}}{C_{i}-\frac{\Gamma *\left[J_{F}+8*\left(P_{N}+R_{d}\right)\right]}{J_{F}-4*\left(P_{N}+R_{d}\right)}}$$

The CO_2_ compensation point to photorespiration (Γ*) was calculated using the RubisCO specificity factor of Galmes et al. [61]. The Kok [62] method was used to estimate respiration in the light (*R*_d_) (PPFD levels of 200, 100, 80, 60, 30, 0 μmol m^−2^ s^−1^) on the 2nd and 12th leaf positions of three plants per treatment and then an average *R*_d_ value was applied to all leaves along the branch. The PSII electron transport rate (*J*_F_) was calculated from chlorophyll fluorescence as

(2) $$J_{F}=\mathrm{PPFD}*\Phi \mathrm{PSII}*\alpha *\beta$$

where the partitioning factor between photosystems I and II was considered as 0.5 (β), leaf absorbance (α) was assumed to be 0.85 [63], and the actual quantum efficiency of PSII (ΦPSII) was determined as

(3) $$\Phi \mathrm{PSII}=\frac{{F_{m}}^{\prime }-F_{s}}{{F_{m}}^{\prime }}$$

where *F*_m_’ is the maximal fluorescence and *F*_s_ is the steady-state fluorescence under light-adapted conditions [64]. Total conductance to CO_2_ (*G*_tot_) was calculated as [65]

(4) $$G_{tot}=\frac{G_{s}*G_{m}}{G_{s}+G_{m}}$$

### 4.3. Leaf Sampling, Measurement of Leaf Water Potential, and Sap Collection

A Scholander pressure chamber (SKPM1400, Skye Instruments, Llandrindod Wells, United Kingdom) was used to measure the water potential of the leaves (*Ψ*_leaf_) used for gas exchange along the largest branch of the six well-watered control and six drought plants after 5 days. On the evening of the fifth day of the experiment, the leaves used for measurement of gas exchange on the largest branch of six plants per water treatment were destructively sampled. The leaves were sampled in the evening to ensure that they contained sugars synthesised during the day; the concentration of sugars are generally lower in the morning due to metabolic and transport processes that occur over the night [53]. The Scholander pressure chamber was used to extract sap using a micropipette from the leaf petiole (Figure 1, point B) of drought-stressed and well-watered control plants after five days. After measurement of *Ψ*_leaf_ and collection of sap from the leaf petiole, the leaves and sap samples were frozen in liquid nitrogen before being stored at −80 °C prior to the extraction and analysis of leaf sugars. Sections of the branch stem of 1 cm in length were collected at the tip, middle, and base of the branch (Figure 1, point C); these were placed into microtubes with ultra-pure water and incubated at 4 °C for 2 h, after which bark rings were removed and the liquid was frozen at −80 °C before purification and isotopic analysis of sugars.

### 4.4. Analysis of Carbon Isotopic Composition and Calculation of G_m_

A leaf disk was removed from the central area of the leaf where gas exchange and Chl-Flr analysis was performed. The disks were ground in liquid nitrogen and shaken for 60 min in water at room temperature. After centrifugation (15 min at 5000 × *g*), the supernatant was sequentially mixed with cationic (Dowex-50) and anionic (Dowex-1) exchange resins. The residual solution of purified soluble sugars was freeze-dried and *δ*^13^C was determined using a continuous-flow triple-collector isotope ratio mass spectrometer (ISOPRIME, GV, Manchester, United Kingdom). The same procedure was used for purification of sugars extracted from leaf petiole and bark tissues. Calculations of carbon isotope discrimination (Δ^13^C) were undertaken following the protocol of Farquhar et al. [66], assuming the carbon isotopic composition of CO_2_ in air (*δ*_air_) to be −8.0‰. The Δ^13^C of recently synthesized sugars method to estimate *G*_m_ utilised the difference between Δ^13^C of leaf soluble carbohydrates (Δ_obs_) and Δ^13^C expected on the basis of gas-exchange measurements (Δ_exp_) [53]:

(5) Gm=(b−bs−a1)∗PNCa(Δexp−Δobs)−(fΓ*/pCO2)

where *b* is the discrimination associated with carboxylation reactions, taken to be 27.5‰; *b*_s_ is the fractionation occurring when CO_2_ enters solutions (1.1‰ at 25 °C); *a*_1_ is the fractionation during diffusion in water (0.7‰); *f* is the fractionation associated with photorespiration, taken to be 0‰ [53,67]; and pCO_2_ is the partial pressure of CO_2_ in air.

## 5. Conclusions

To the best of our knowledge, the present study represents the first experimental analysis of average *G*_m_ integrated at leaf and branch level in water-stressed plants using the approach of Ubierna and Marshall [46]. Drought resulted in pronounced reductions in the conductance of CO_2_ across the mesophyll layer of cherry (Figure 2 and Table 1). This was likely associated with reduced photosynthetic CO_2_ assimilation and lower *G*_s_. The variable *J* and C-isotopic composition of sugars within the leaf produced the most comparable estimate in terms of absolute values of *G*_m_ (Figure 5a). This correspondence is likely due to the sugars within the leaf being the most recently synthesised, and thus most closely reflecting the diffusive limitations and C-isotopic discrimination conditions captured in the instantaneous variable *J* measurements. The higher *δ*^13^C of sugars from the leaf petiole may reflect further post-photosynthetic fractionation processes in favour of ^12^C by metabolic processes within the leaf resulting in enrichment of ^13^C in sugars in the leaf petiole, and, therefore, producing lower estimates of *G*_m_ (Figure 5b). Average branch canopy *G*_m_ estimated from the sugars of branch sap were more variable under drought and control conditions than the other protocols. This may have been due to limitations in utilising gas exchange measurements of individual leaves [68] when scaling-up to estimate *G*_m_ on the basis of the C-isotopic composition of branch sap sugars, which reflect larger temporal and spatial effects of photosynthetic and post-photosynthetic C-isotopic fractionation processes and the influence of environmental factors. This is particularly relevant in terms of the effects of short-term changes of environmental conditions, such as the intense drought event encapsulated within the present study. In effect, the further away from the source of sugars in the leaves, the less robust the correlation and correspondence in absolute values of *G*_m_ to those produced by the variable *J* method. Nonetheless, the variable *J* and C-isotopic analysis of sugars methods produced broadly similar estimates of *G*_m_, suggesting that both methods may be effective and complementary in the field and laboratory. However, when measuring *G*_m_, attention should be given to the time frame and the most appropriate scale of analysis (individual leaves or average canopy) of *P*_N_ with respect to the proposed dynamics of the experimental treatment or environmental variations under consideration. The methodology must be suited to the aims of the study with respect to temporal and spatial variation in *G*_m_.

## Figures and Tables

**Table 1:** *δ*^13^C values of sugars extracted from the leaf, leaf petiole sap, and branch sap (Figure 1), and *G*_m_ estimates from the *δ*^13^C of those sugars and the variable *J* method in control and drought-stressed cherry seedlings after 5 days. Upper case superscript letters indicate homogenous groups in *δ*^13^C of sugars and superscript lower case letters indicate homogenous groups in estimates of *G*_m_ determined using a one-way ANOVA with a Fisher’s Least Significant Difference (LSD) post-hoc test. Values are the means ± standard error. Degrees of freedom for leaf and leaf petiole measurements are F_1,34_ and for branch measurements F_1,5_.

	Sugar *δ*^13^C (‰)		*G*_m_ C-Isotopic Sugars (mol m^−2^ s^−1^ bar^−1^)		*G*_m_ Variable *J* (mol m^−2^ s^−1^ bar^−1^)	
	*Control*	*Drought*	*Control*	*Drought*	*Control*	*Drought*
**Leaf**	−24.700 ± 0.178^C^	−23.355 ± 0.189^B^	0.333 ± 0.045^a^	0.039 ± 0.010^d^	0.278 ± 0.038^ab^	0.021 ± 0.008^d^
**Leaf petiole sap**	−23.166 ± 0.146^B^	−22.500 ± 0.132^A^	0.228 ± 0.031^b^	0.026 ± 0.006^d^	-	-
**Branch sap**	−24.427 ± 0.116^C^	−23.450 ± 0.199^B^	0.287 ± 0.054^ab^	0.125 ± 0.011^c^	-	-
